# Paul W. Turke, Review of Bringing Up Baby: An Evolutionary View of Pediatrics

**DOI:** 10.1093/emph/eoaf007

**Published:** 2025-02-27

**Authors:** Bernard Crespi

**Affiliations:** Department of Biological Sciences, Simon Fraser University, Burnaby, British Columbia, V5A 1S6, Canada

My wife and I were quietly horrified, as we walked out of the hospital with our first newborn, that we were actually being allowed to leave. Didn’t anyone realize that we had vanishingly little idea of how to look after a baby? We needed help, a *focus*. Being scientists, we latched onto the well-organized printouts that the nurses had provided, to record the timing of inputs (breast milk) and outputs (use your imagination). If only we had, back then, this book.

*Bringing Up Baby* represents the dawn of Darwinian pediatrics, or at least its cries in the early morning. Its author Paul Turke, an exceptional mix of medical doctor and evolutionary anthropologist, has written a superb book that shines evolutionary light on some of the major challenges of successfully raising a child from birth to adolescence. The book is aimed at the general public, but it is stimulating for specialists such as the readers of *Evolution, Medicine, and Public Health*. It begins with a hilarious account of Dr. Turke’s transition to medical school, where he is told by a medical professor: ‘you’re thinking like a scientist; I’m not sure that’s appropriate for a medical student’ (leaving open the question of how scientists and doctors should think). The book continues with chapters on sleeping and breastfeeding, immunity, disease and vaccination, diet and food allergies, injury and pain, and mental illness with emphasis on depression—core elements of the curriculum that my wife and I needed so desperately. It finishes with chapters on sex and reproduction, and human lifespan in the context of cooperative childcare, thus, at the end, returning to the evolutionary anthropology where it all began.

The book is delightfully well written, with a series of vignettes, collected from a long career in the trenches of meeting pediatric patients and their caregivers, that serve to illustrate key points about adaptation and disease. And, of course, about the medical system, and doctors, and how they are trained, or, better put, indoctrinated. In this context, anthropologist Paul Turke among the pediatricians is not nearly Napolean Chagnon among the Yanomamo (Chagnon was his adviser), but he provides unique perspectives and insights that deeply illuminate the gulfs between doctors and evolutionary biologists. Both scientists, and MDs, should benefit from these perspectives in an enhanced appreciation of how the ‘other half’ thinks and acts. Perhaps most importantly, readers with neither medical nor scientific backgrounds (most of humanity, of course) will gain from better understanding of evolutionary biology, human health professions, and how to best rear our children—a goal shared by everyone.

This, after all, is the biggest challenge of evolutionary medicine: changing how people think about disease and well-being. In this regard, *Bringing Up Baby* does an admirable job of explaining key ideas such as adaptation (simple in principle, nuanced in application), natural selection, and the relationships of health and happiness with fitness and reproduction. An even more difficult, yet equally important task is evidential: how is a non-scientific reader to know what to believe, who to trust: one’s own doctor, one’s news feed, a government agency, the first-page results of a Google search, or, better yet, this book? More background on hypothesis testing would have helped here; after all, one of the main goals of evolutionary medicine is the formulation of novel, testable hypotheses, based on ideas including mismatches, conflicts, trade-offs, extremes of adaptations, and constraints, that would likely never have entered the brains of non-evolutionary medical researchers or practitioners.

We need 10,000 young Paul Turkes, in diverse fields of medicine, to most directly and efficiently drive forward the integration of evolutionary biology with health research and practice, with direct impacts on the welfare of our children. Medical schools could provide them, in principle. But if history is our guide, and as this book shows all too well, the staunch conservatism of medicine, and the mind-sets of most doctors, mean that this will happen too slowly if at all. Instead, practitioners and promoters across all fields of evolutionary medicine might usefully consider writing their own popular, how-to, learn-it-yourself books for the educated public, on the evolutionary medicine of obesity, cancer, exercise, pain and injury, autism, infectious disease, endometriosis, immunity, heart disease, stroke, senescence, or any other topic of interest. Make the book rigorous yet engaging, and short but comprehensive enough to be practical and useful for informed decision-making. *Bringing Up Baby* can serve as a model and inspiration. After all, how many people, real people with young babies or medical challenges, will read your scientific articles?

